# Supplementary material for: FOXA2 potently represses NF-κB–dependent transcription from viral promoters in liver cells
Source: J Biol Chem. 2026 Jul 28;302(9):113369. doi: 10.1016/j.jbc.2026.113369 (PMC13543952; doi:10.1016/j.jbc.2026.113369)
Supplement: Supplementary file 1 [file mmc1.pdf]

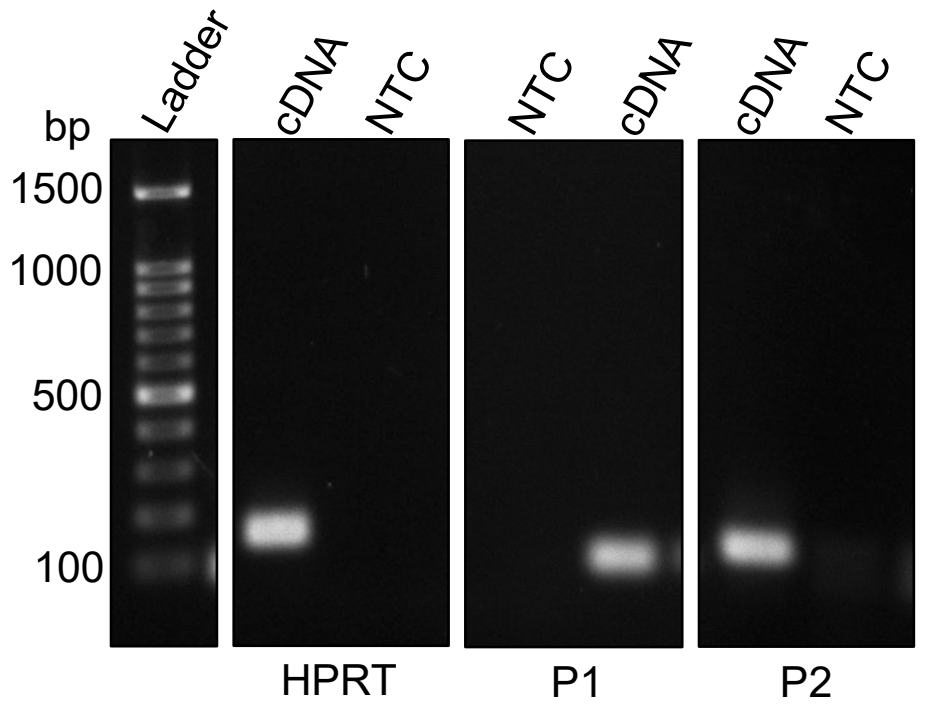

Figure S1. Transcript-specific RT-PCR confirms endogenous expression of both FOXA2 transcript variants in HepG2 cells. RT-PCR was performed using transcript-specific primer pairs designed to selectively amplify FOXA2-P1- and FOXA2-P2-derived transcripts. PCR products were resolved on a 1% agarose gel alongside a 100-bp DNA ladder. Expected amplicon sizes were 78 bp (P1), 110 bp (P2), and 139 bp (HPRT). HepG2 cDNA was used as template for experimental samples. HPRT was amplified as a control for cDNA integrity and successful reverse transcription. No amplification was observed in no-template control (NTC) reactions in which water was substituted for cDNA. The gel was cropped to remove irrelevant lanes for clarity.

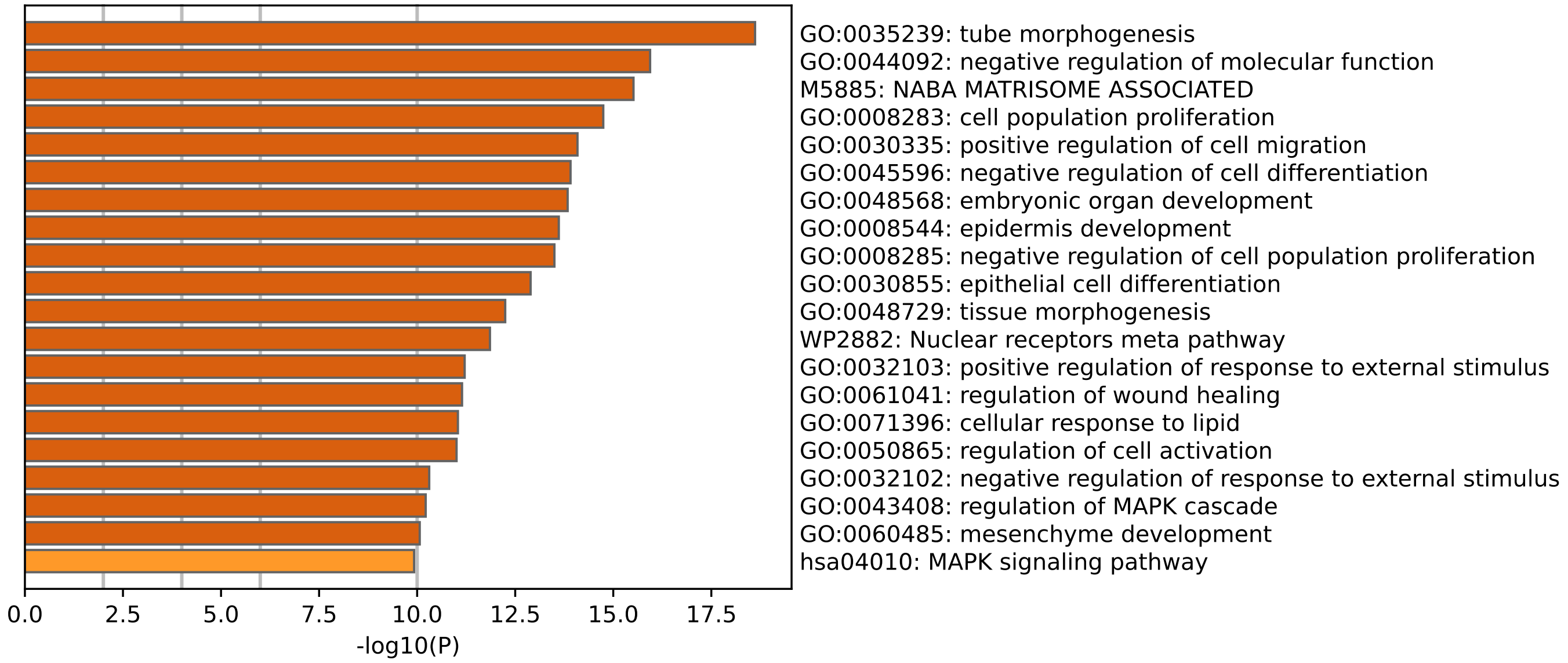

Figure S2. Enriched pathways in HepG2 cells following FOXA2 over-expression. Gene Ontology (GO) enrichment analysis of downregulated genes in HepG2 cells upon over-expression of FOXA2. Bar graphs represent significantly enriched biological processes, colored according to adjusted p-values, as determined by Metascape.

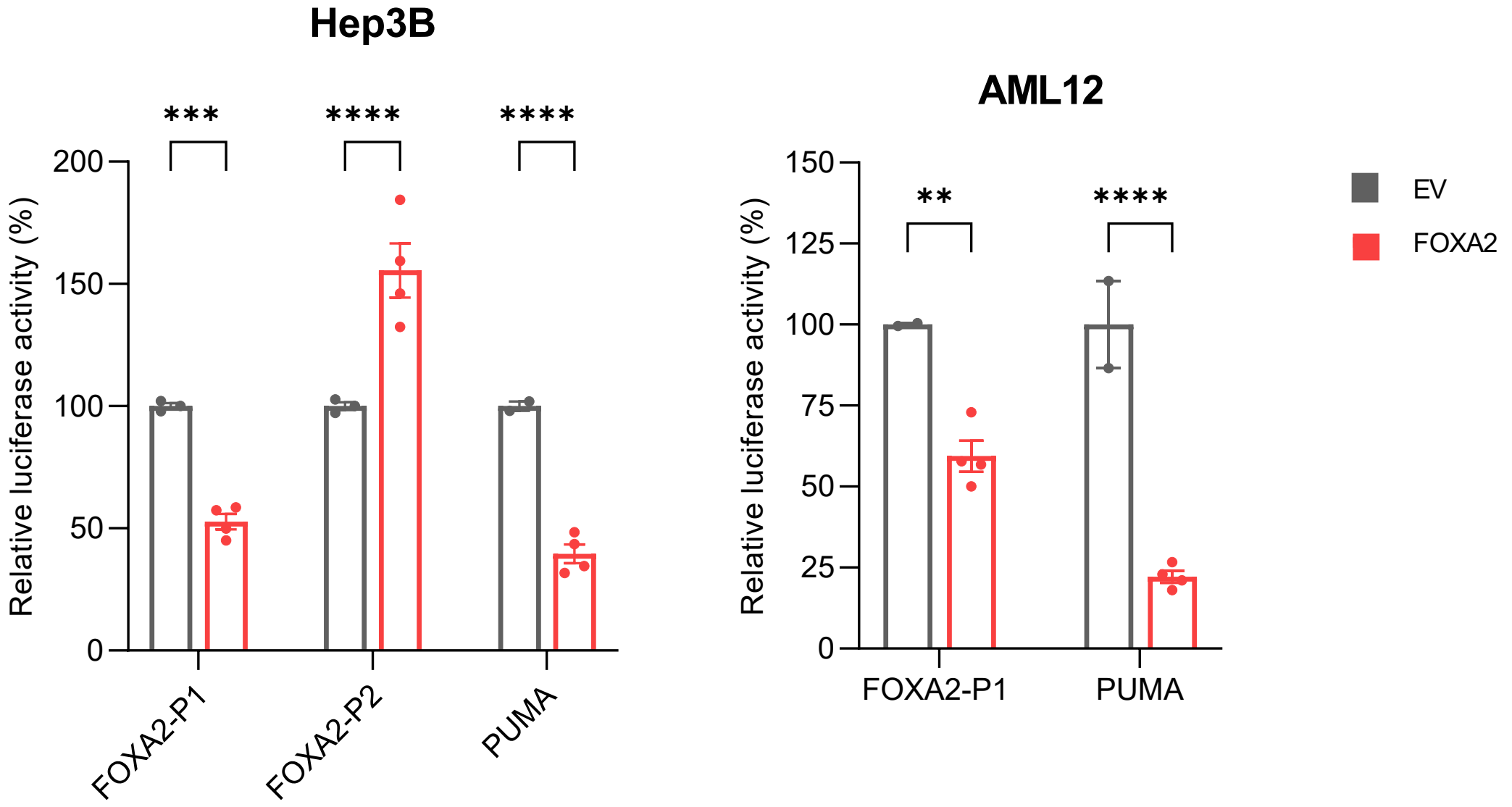

Figure S3. Repression of selected cellular promoters by FOXA2 in additional liver cell lines. Additional liver-derived cell lines were transiently transfected with 200 ng of plasmids containing the indicated cellular regulatory elements upstream of a firefly luciferase reporter gene. Cells were co-transfected with 350 ng of either empty vector (EV; control) or a plasmid encoding FOXA2-P1. Results are presented as mean ± SEM for two independent experiments with two biological replicates per experiment. Statistical analysis was performed using ordinary two-way ANOVA with Sidak's multiple comparisons test (****$p < 0.0001$, ***$p < 0.001$, **$p < 0.01$).

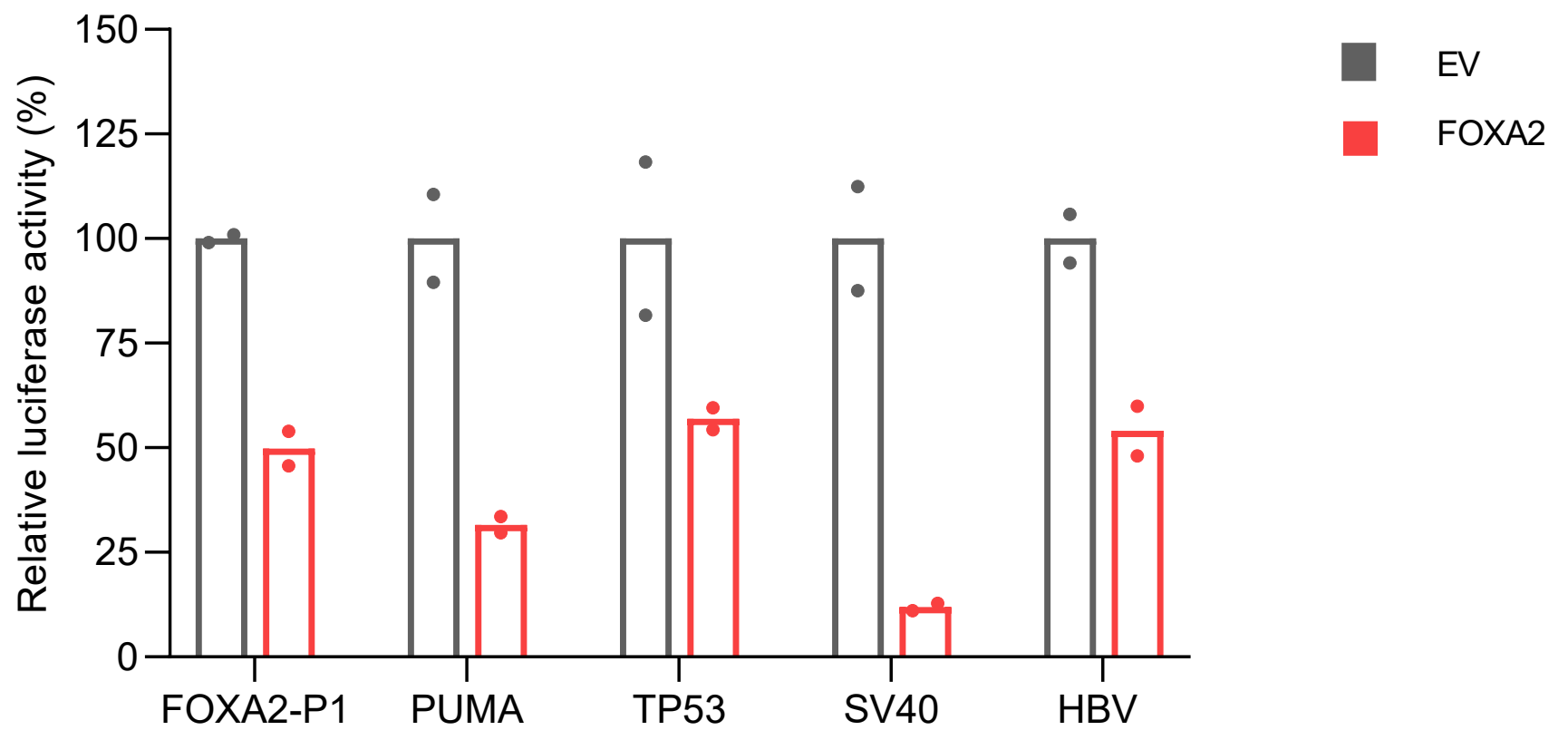

Figure S4. Repression of selected promoters by FOXA2 is recapitulated upon RSV-driven FOXA2 expression in HepG2 cells. Cells were transiently transfected with 250 ng of plasmids containing the indicated cellular or viral regulatory elements upstream of a firefly luciferase reporter gene, and co-transfected with 300 ng of either empty vector (EV; control) or a plasmid encoding FOXA2-P1 under the RSV promoter. Data are shown as mean values from two biological replicates.

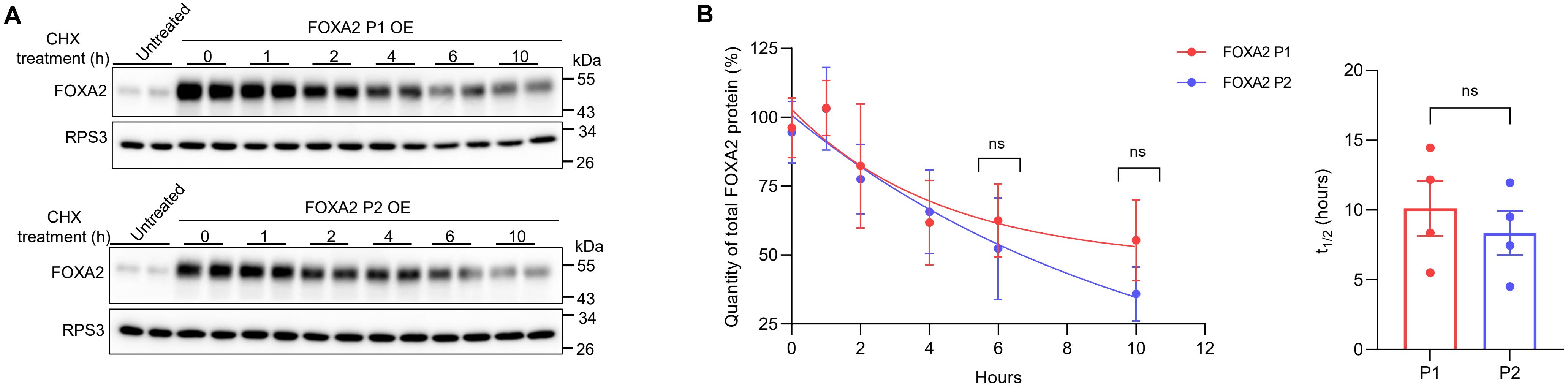

Figure S5. Cycloheximide pulse-chase analysis of FOXA2 isoform stability in HepG2 cells. After transient transfection of HepG2 cells with expression plasmids encoding FOXA2 protein isoforms, cells were treated with cycloheximide and harvested at the indicated time points.

(A) Western blot analysis of FOXA2 P1 (upper) and P2 (lower) using 7.5 µg of total protein lysate per lane, with RPS3 used as a loading control. Estimated molecular weights are indicated. “Untreated” denotes control samples not transfected with FOXA2-expressing plasmids. The blot shows biological replicates from a single experiment, which was independently repeated four times.

(B) Left: relative quantification of FOXA2 protein levels normalized to RPS3. Data points represent mean ± SD. Right: calculated protein half-lives based on decay curves. Protein half-lives ($t_{1/2}$) were calculated using the equation: $t_{1/2} = \frac{\ln 2}{k_{decay}}$, where the decay constant (k>0) was obtained by exponential decay fitting of normalized protein levels as a function of time. The estimated half-life was 10.1 h for the P1 isoform and 8.3 h for the P2 isoform. Results are presented as mean ± SEM from four independent experiments with two biological replicates each. Statistical analysis was performed using unpaired two-tailed Student’s t test (ns, $p > 0.5$).

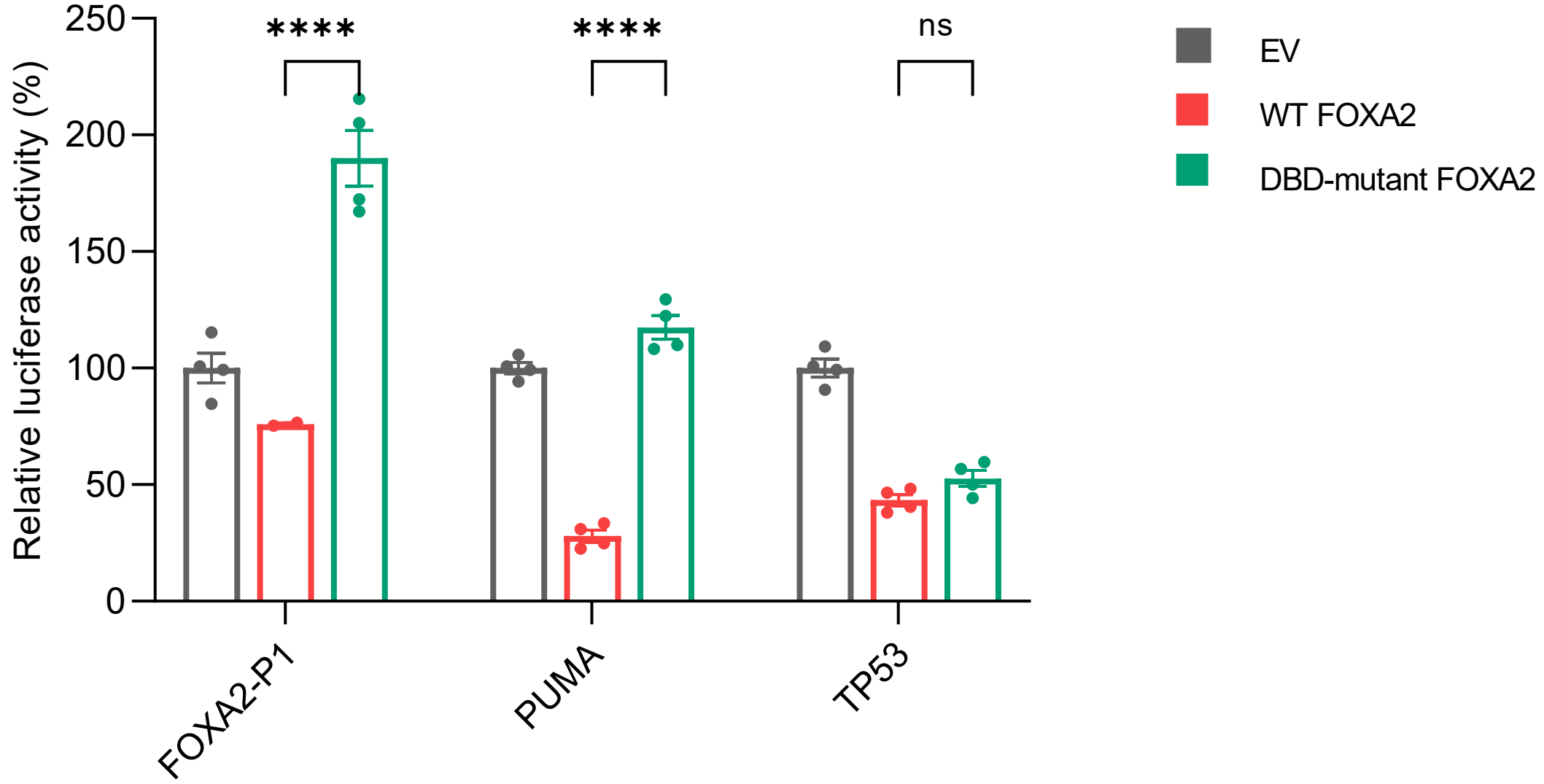

Figure S6. DNA binding by FOXA2 is required for repression of a subset of cellular regulatory elements. Transactivation assays of cellular regulatory elements by wild-type and DBD-mutant FOXA2. HepG2 cells were transiently transfected with 200 ng of plasmids containing the indicated cellular promoters upstream of the firefly luciferase reporter gene. In addition, 90–275 ng of either a plasmid encoding FOXA2 P1 or a plasmid encoding DBD-mutant FOXA2 P1 were co-transfected and compared with samples transfected with noncoding empty vector (EV) alone. The same total amount of DNA was used in all transfections (with EV used as a carrier to reach 600 ng when required). Results are presented as mean ± SEM from two independent experiments with two biological replicates each. Statistical analysis was performed using ordinary two-way ANOVA with Tukey's multiple comparisons test (****$p < 0.0001$, ns $p > 0.5$).

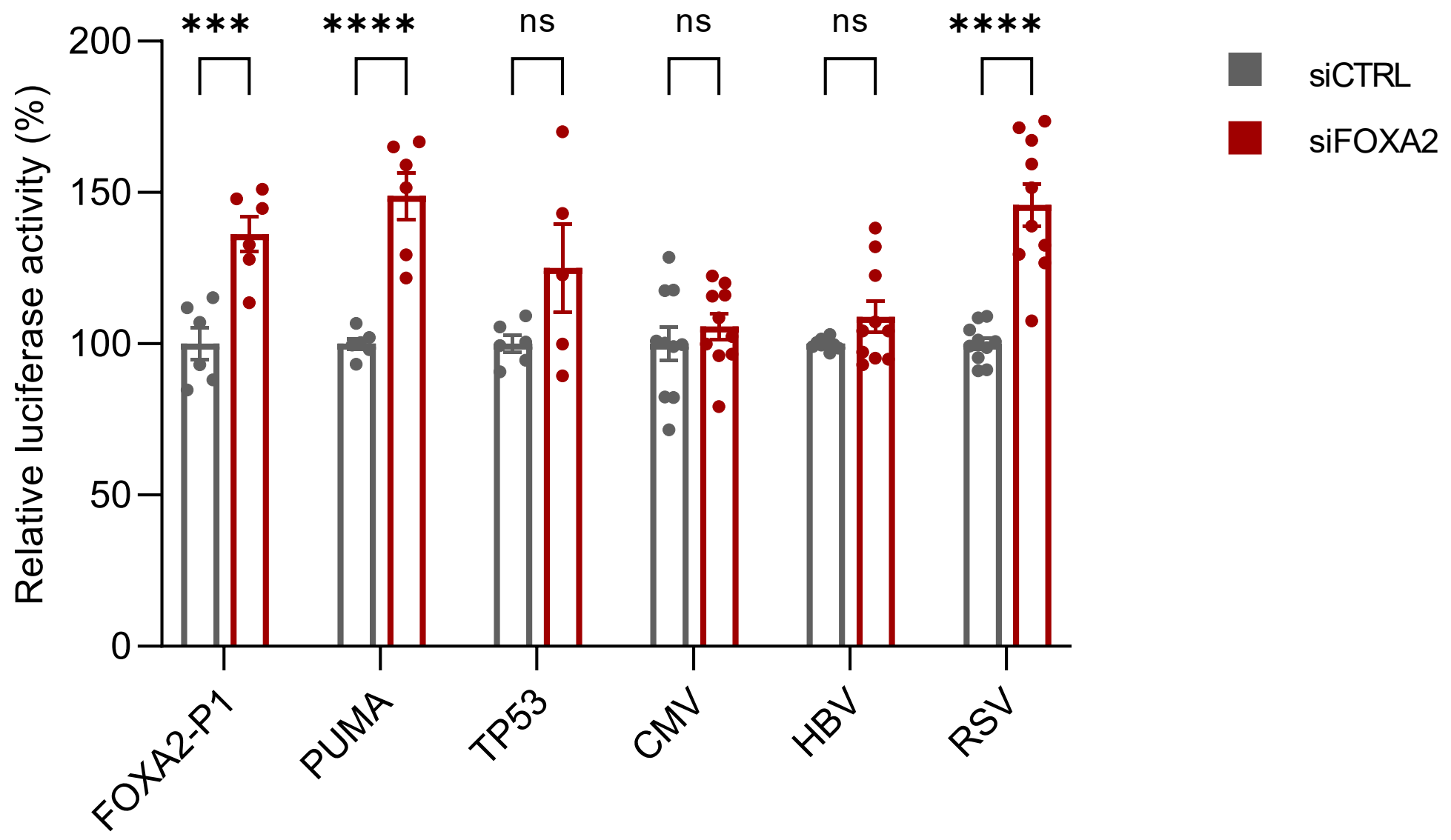

Figure S7. FOXA2-mediated repression of most tested regulatory elements is partially relieved by knockdown. HepG2 cells were transiently transfected with either siRNA targeting FOXA2 (siFOXA2) or a non-targeting control siRNA (siCTRL). Cells were subsequently transfected with 250 ng of firefly luciferase reporter plasmids driven by the indicated cellular or viral elements, together with 250 ng empty vector (EV). Results are presented as mean ± SEM from three (for cellular elements) or five (for viral elements) independent experiments, each performed with two biological replicates. Statistical analysis was performed using ordinary two-way ANOVA with Sidak's multiple comparisons test (****$p < 0.0001$, ***$p < 0.001$, ns $p > 0.05$).

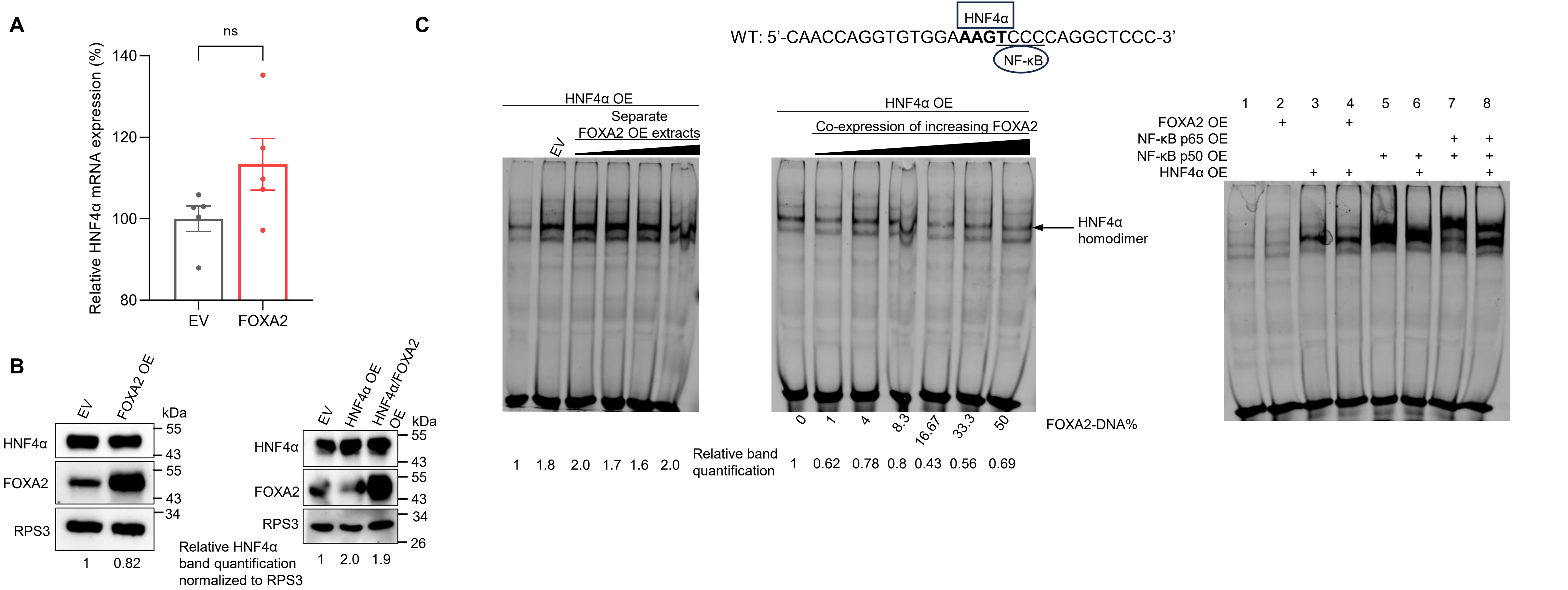

Figure S8. FOXA2-mediated repression of the SV40 early promoter is independent of HNF4α.
(A) Relative expression levels of the endogenous HNF4α gene derived from RNA-seq analysis. Results are presented as mean ± SEM from five biological replicates. (ns $p > 0.5$)
(B) Western blot analysis of whole-cell (left) and nuclear (right) extracts from HepG2 cells following transfection with FOXA2 (left) or HNF4α, with or without FOXA2 co-expression (right), as indicated. A total of 10 µg of nuclear protein were loaded per lane, whereas 5 µg of whole-cell extract were loaded. RPS3 was used as a loading control. Estimated molecular weights are indicated.
(C) EMSA showing protein–DNA complexes in nuclear extracts from HepG2 cells following over-expression of HNF4α and NF-κB, as indicated. The transcription factor binding sites present in the oligonucleotide used for EMSA are shown in the upper panel; NF-κB and HNF4α motifs identified using the Genomatix Genome Analyzer (high-confidence binding defined as core ≥ 0.9 and matrix ≥ 0.85). **Lower left panel:** In the first lane, 5 µg of nuclear extract from HNF4α-over-expressing cells was loaded alone. In subsequent lanes, these extracts were mixed with separate nuclear extracts from cells transfected with empty vector (EV) or increasing amounts of FOXA2. Equal amounts of protein from each extract (5 µg + 5 µg) were combined prior to EMSA. **Lower middle panel:** EMSA showing HNF4α–DNA complexes in nuclear extracts from HepG2 cells following co-expression of increasing amounts of FOXA2 (transfected DNA percentages shown below the gel) together with constant amounts of HNF4α, as indicated. In all lanes, 5 µg of nuclear extract was loaded. **Lower right panel:** EMSA showing binding of NF-κB and HNF4α to DNA under the different co-expression conditions indicated above the gel. Results are representative of two independent experiments.

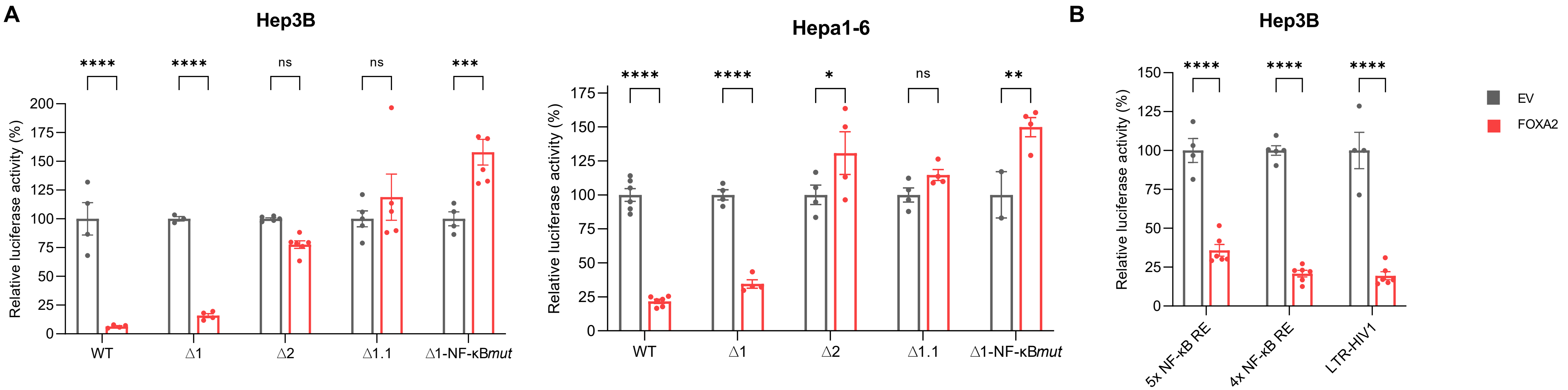

Figure S9. FOXA2-mediated repression of SV40 promoter mutants and NF-κB–dependent reporter constructs across liver-derived cell lines.

(A) Transactivation assays of wild-type and mutant SV40 early promoter constructs by FOXA2. Liver-derived cell lines were transiently transfected with 200 ng of plasmids containing either wild-type or mutant SV40 early promoter constructs upstream of the firefly luciferase reporter gene. Cells were co-transfected with 350 ng of a plasmid encoding FOXA2 or noncoding empty vector (EV) as control. Results are presented as mean ± SEM from two independent experiments with two-three biological replicates each. Statistical analysis was performed using ordinary two-way ANOVA with Sidak’s multiple comparisons test (****$p < 0.0001$, ***$p < 0.001$, **$p < 0.01$, *$p < 0.05$, ns $p > 0.05$).

(B) Transactivation assays of NF-κB–dependent constructs by FOXA2 in Hep3B cells. Similar transfection conditions and statistical analyses were used as described in (A).

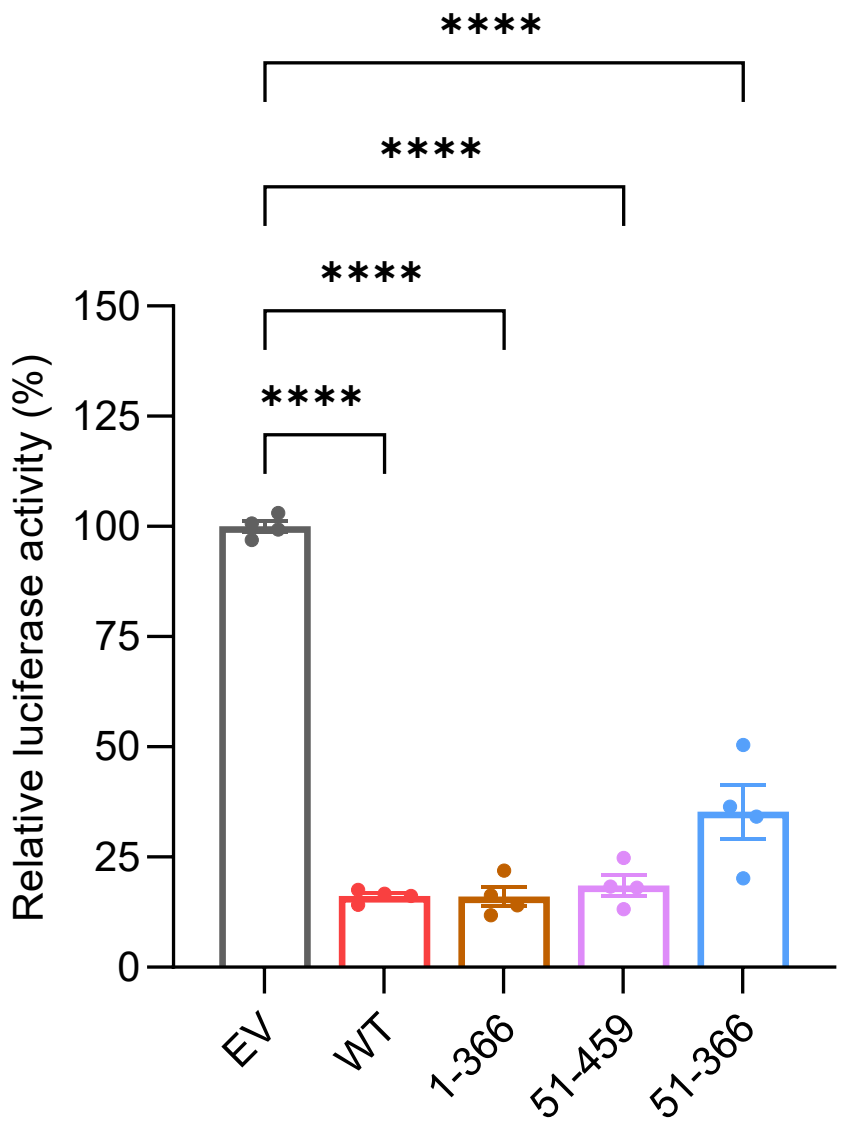

Figure S10. FOXA2 DNA binding domain mediates repression of the 5×NF-κB response element reporter in HepG2 cells. Transactivation assays of a 5×NF-κB response element by wild-type and truncated variants of FOXA2. HepG2 cells were transiently transfected with 200 ng of a plasmid containing the 5×NF-κB response elements upstream of the firefly luciferase reporter gene, together with 350 ng of either noncoding empty vector (EV) or plasmids encoding wild-type or truncated variants of FOXA2 P1. Results are presented as mean ± SEM from two independent experiments with two biological replicates each. Statistical analysis was performed using ordinary one-way ANOVA followed by Tukey's multiple comparisons test (****$p < 0.0001$).

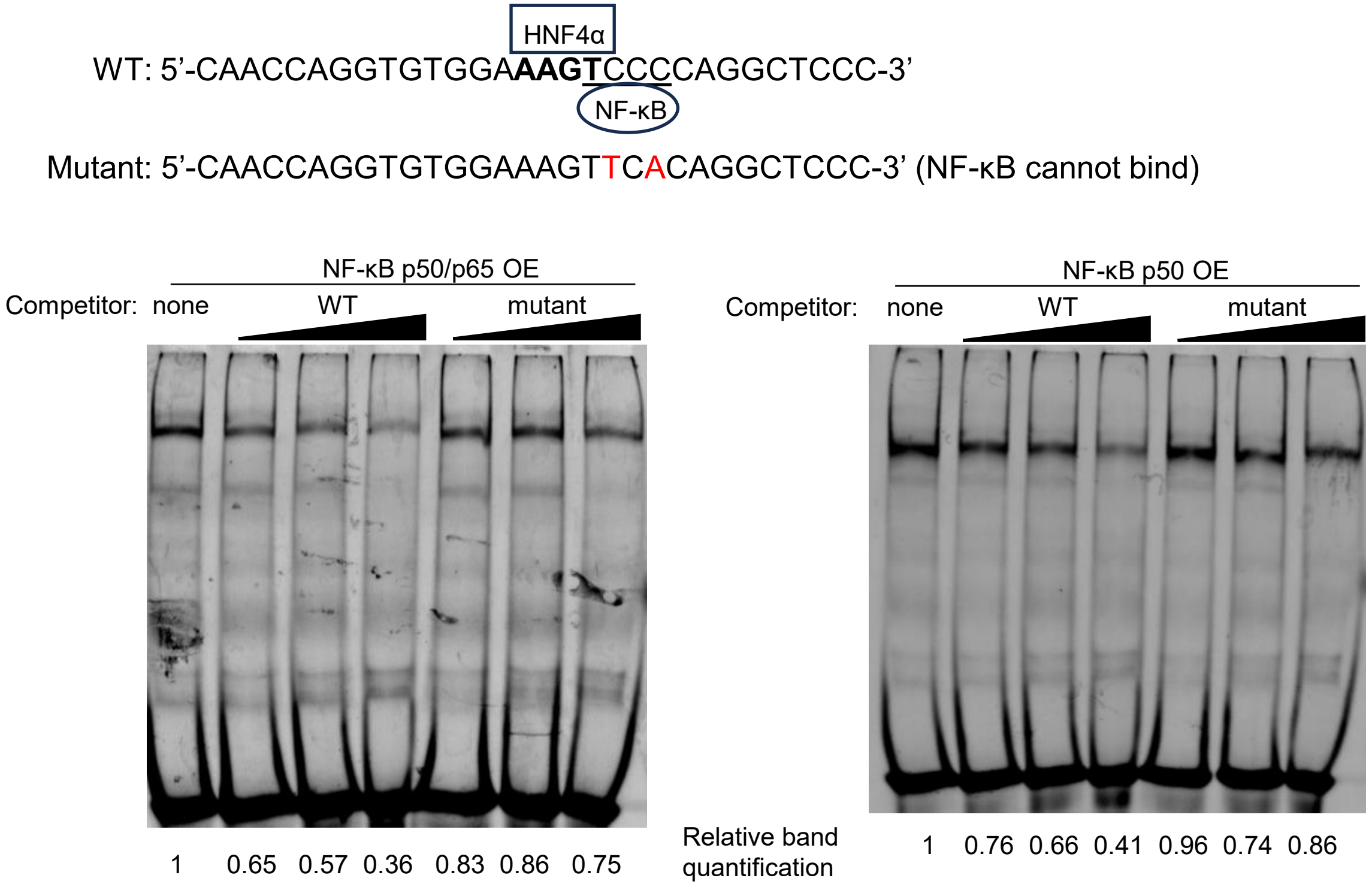

Figure S11. Validation of NF-κB identity in EMSA by competition with wild-type and mutant oligonucleotides. Upper panel shows the sequences of wild-type and mutant oligonucleotides used as competitors. Lower panel shows EMSA of NF-κB–DNA complexes using nuclear extracts from cells over-expressing p50/p65 (left) or p50 alone (right). In the first lane, labeled wild-type probe was used alone. In subsequent lanes, increasing concentrations (2.5, 5, and 10 nM) of unlabeled wild-type or mutant competitor oligonucleotides were included in the binding reactions; the amount of labeled probe was identical in all samples. Five µg of nuclear extract were loaded per lane. Band quantification demonstrates specific competition of NF-κB–DNA complexes by wild-type but not mutant oligonucleotides.

A

Sample	Adjusted band intensity
NF-κB p65/p50 OE	94,677,912
NF-κB p65/p50 OE + EV	97,978,860
NF-κB p65/p50 OE + extracts with increasing FOXA2	101,544,408
	96,713,568
	99,193,752
	100,314,774

Sample	Adjusted band intensity
NF-κB p50 OE	216,228,593
NF-κB p50 OE + EV	234,011,834
NF-κB p50 OE + extracts with increasing FOXA2	262,552,318
	223,597,430
	217,708,834
	175,372,561

B

FOXA2 plasmid DNA (%)	Adjusted band intensity
0	48,381,760
16.67	40,655,494
66.67	14,680,512

FOXA2 plasmid DNA (%)	Adjusted band intensity
0	110,210,125
1	70,254,375
4	57,711,750
8.3	54,968,625
16.67	33,395,375
33.3	37,216,750
50	23,102,625

C

			NF-κB p65		NF-κB p50	
Sample	RPS3	Normalizing factor	Band intensity	Normalized	Band intensity	Normalized
EV	80,801,072	1.0	14,195,230	14,195,230	4,414,496	4,414,496
FOXA2 OE	91,332,639	0.9	13,151,648	11,635,131	2,366,520	2,093,637

D

			NF-κB p65		NF-κB p50	
Sample	RPS3	Normalizing factor	Band intensity	Normalized	Band intensity	Normalized
NF-κB OE	61,441,900	1.0	34,325,953	34,325,953	85,146,950	85,146,950
NF-κB OE + MG132	26,778,944	2.3	45,635,040	104,705,531	87,558,950	200,896,206
NF-κB & FOXA2 OE	42,251,310	1.5	20,146,797	29,297,494	34,937,284	50,805,836
NF-κB & FOXA2 OE + MG132	62,659,564	1.0	33,045,421	32,403,249	63,025,426	61,800,652

Figure S12. Quantification of EMSA and Western blot bands.

Tables showing densitometric quantification from a representative experiment of the gels shown in the indicated figures: **(A)** EMSA in Fig. 7D; **(B)** EMSA in Fig. 7E (left table) and Fig. 7F (right table); **(C)** Western blot in Fig. 7B; **(D)** Western blot in Fig. 8B.

All densitometric quantification of bands was performed using Image Lab 6.1 software (Bio-Rad). Adjusted band intensity values were obtained by densitometric analysis of EMSA bands following background subtraction. Western blot data were normalized to the indicated loading control (RPS3). Each protein was analyzed on separate immunoblots and quantified independently; as a result, absolute band intensities are not comparable between proteins and only relative changes within each protein are meaningful. Quantifications of other experiments not shown here are available from the authors upon reasonable request.
